# Supplementary material for: Capillary trapping of various nanomaterials on additively manufactured scaffolds for 3D micro-/nanofabrication
Source: Nat Commun. 2024 Aug 6;15:6693. doi: 10.1038/s41467-024-51086-2 (PMC11303746; doi:10.1038/s41467-024-51086-2)
Supplement: Supplementary file 1 — Supplementary Information [file 41467_2024_51086_MOESM1_ESM.pdf]

Supplementary information for

Capillary trapping of various nanomaterials on additively manufactured scaffolds for 3D micro-/nanofabrication

Xianglong Lyu, Zhiqiang Zheng, Anitha Shiva, Mertcan Han, Cem Balda Dayan, Mingchao Zhang*, Metin Sitti*

* Correspondence to: zhangmc@is.mpg.de (M.Z.), sitti@is.mpg.de (M.S.)

The PDF file includes:

Supplementary Figures 1 to 17
Supplementary Tables 1 to 3
Supplementary references

Other Supplementary Materials for this manuscript include the following:

Supplementary Movies 1 to 5

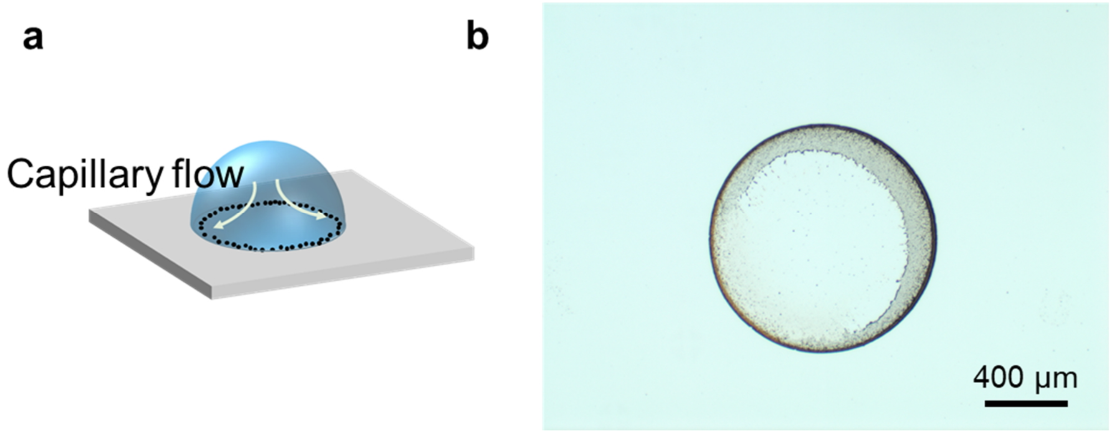

Supplementary Figure 1 Formation of a coffee ring pattern on the substrate. a, Schematic of the formation of a coffee-ring-pattern made of nanomaterials on a substrate. **b**, Optical microscope image of a coffee ring after dropping and drying 2 μL Au NPs aqueous solution (≈4 mg mL^{-1}) on a silane-treated substrate.

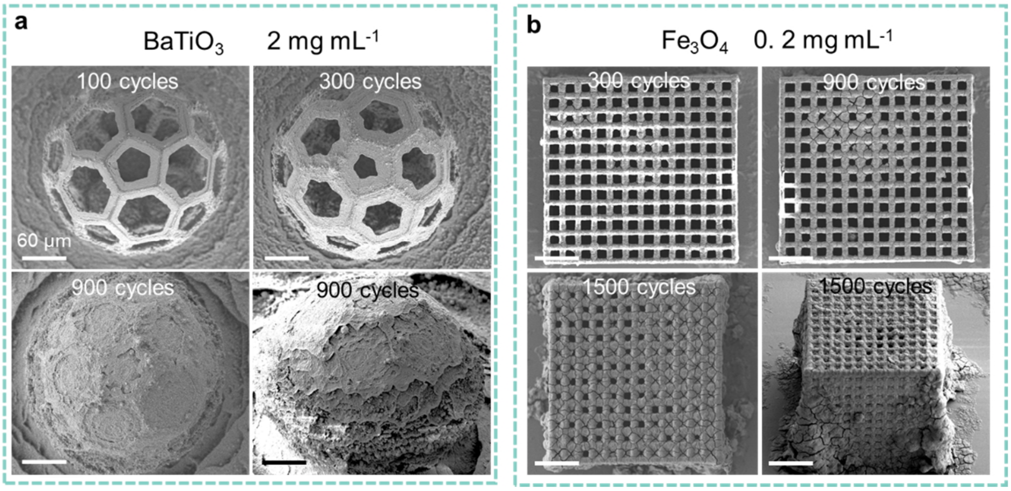

Supplementary Figure 2 a, b, SEM images of a hollow microsphere assembled with $BaTiO_3$ (a) and a cube-shaped microscaffold assembled with Fe_3O_4 (b) for different immersion/retraction cycles.

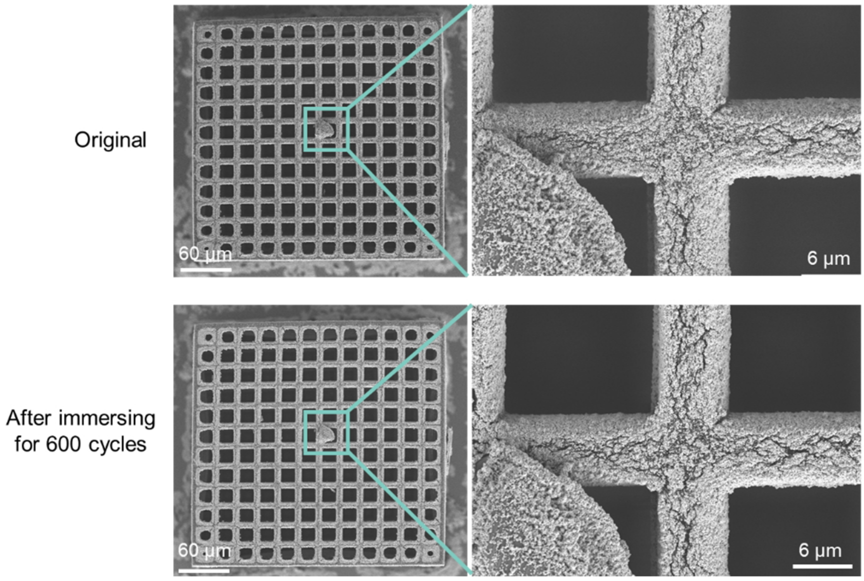

Supplementary Figure 3 SEM images of Au NPs assembled microscaffold before and after 600 immersion/retraction cycles in pure 90% IPA/H_2O solution (no nanoparticles contained). The morphology basically remains unchanged on the structure after immersing, indicating that the assembled particles are robust enough to resist the redispersion in the following immersing process.

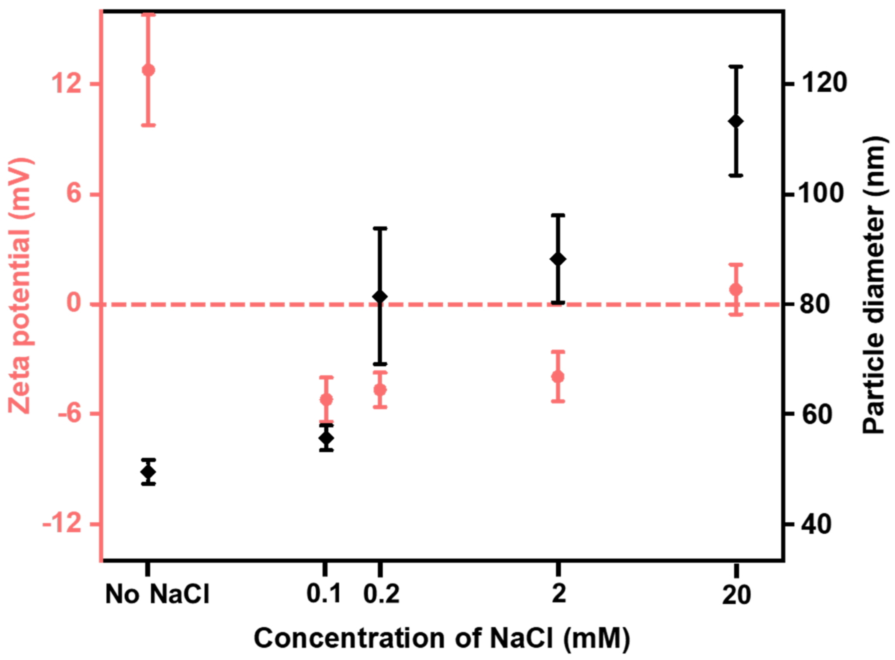

Supplementary Figure 4 Zeta potentials (red circle) and hydrated dimeters (black diamonds) of Au NPs dispersed in 90% IPA/H_2O solutions with different concentrations of NaCl. Source data are provided as a Source Data file. These data points are shown as mean ± s.d. with at least 4 independent measurements. (n≥4).

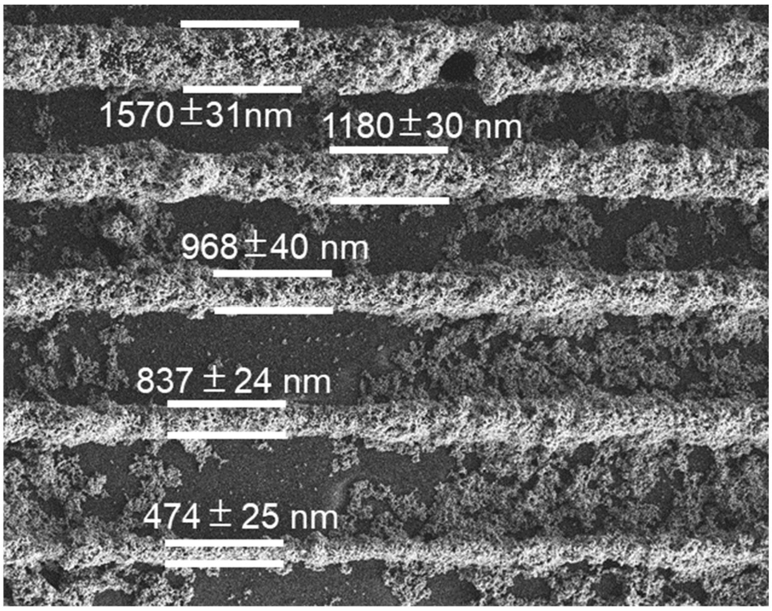

Supplementary Figure 5 SEM image of Au NP-assembled IPS line scaffolds with different widths. As the strategy is based on template-assisted fabrication, the resolution of this method thus depends on that of the 2PP-printed scaffolds (which could be further down to ≈100 nm). Considering the weak trapping capability of lines, the experiment was conducted by dropping and drying 5 μL 90% IPA/H_2O solution with ≈4 mg mL^{-1} Au NP and 0.01 mM PBS for 12 cycles on the silane-treated substrate. Source data are provided as a Source Data file.

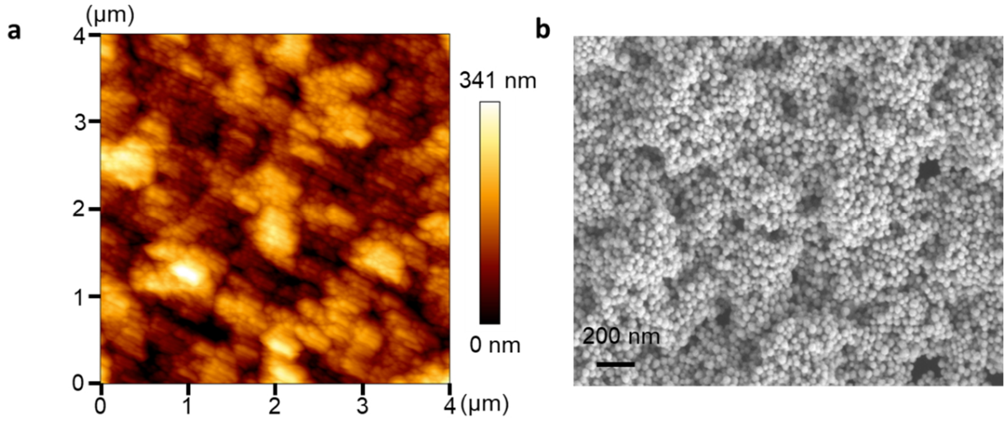

Supplementary Figure 6 Roughness characterization of nanomaterial-assembled structures. a, b, AFM measurement (**a**) and SEM image (**b**) of an Au NPs (40 nm) assembled surface after 900 immersion/retraction cycles.

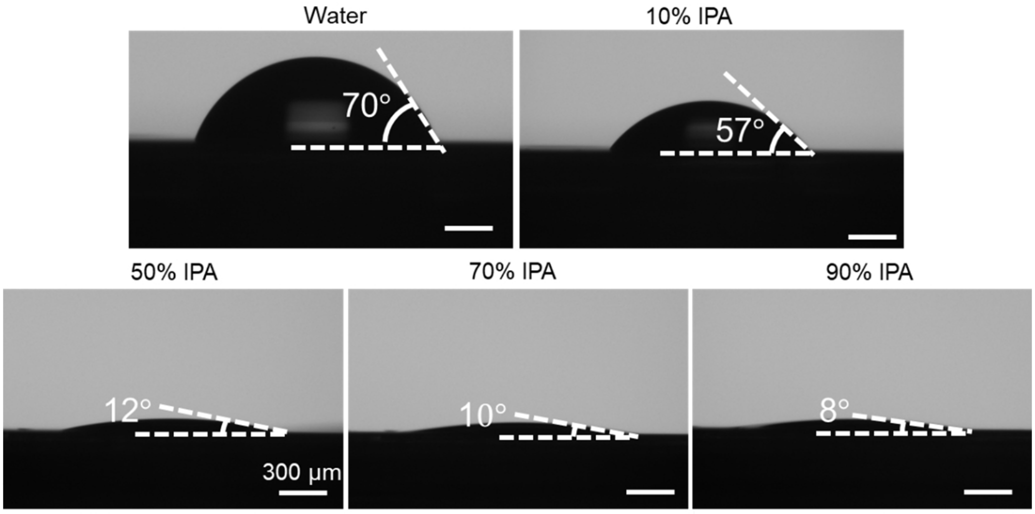

Supplementary Figure 7 Optical images showing the contact angle measurements of solutions containing 0.01 mM PBS solutions with different IPA/H_2O ratio on an IPS film (cross-linked) without any surface treatment.

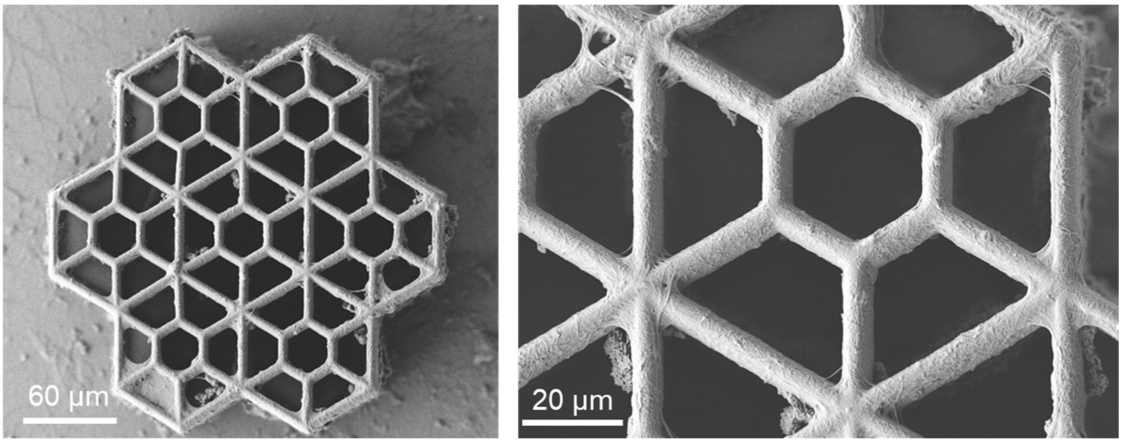

Supplementary Figure 8 SEM images of a honeycomb-like microscaffold assembled with Fe_3O_4 NPs after 1600 immersion-retraction cycles in toluene. Different solvent systems, such as the non-polar solvents, can be employed to achieve similar results.

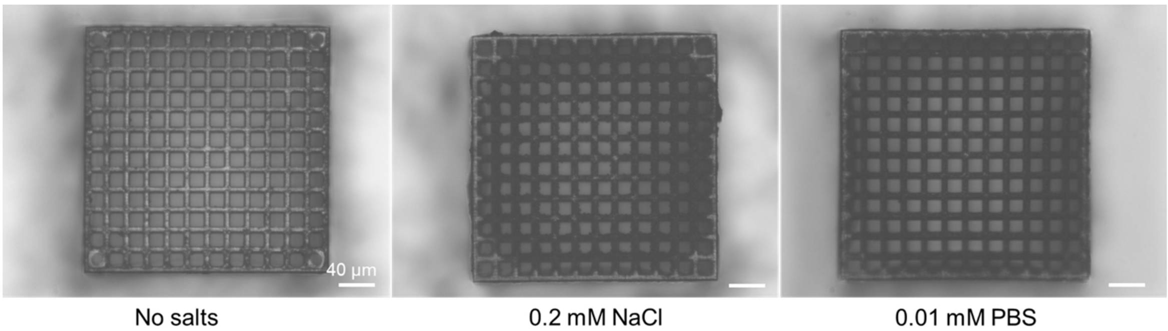

Supplementary Figure 9 8-bit optical images of a microscaffold coated with Au NPs in solutions with and without salts. These experiments are performed in 90% IPA/H_2O solutions with $\approx$1.2 mg mL^{-1} Au NP for 600 immersion-retraction cycles.

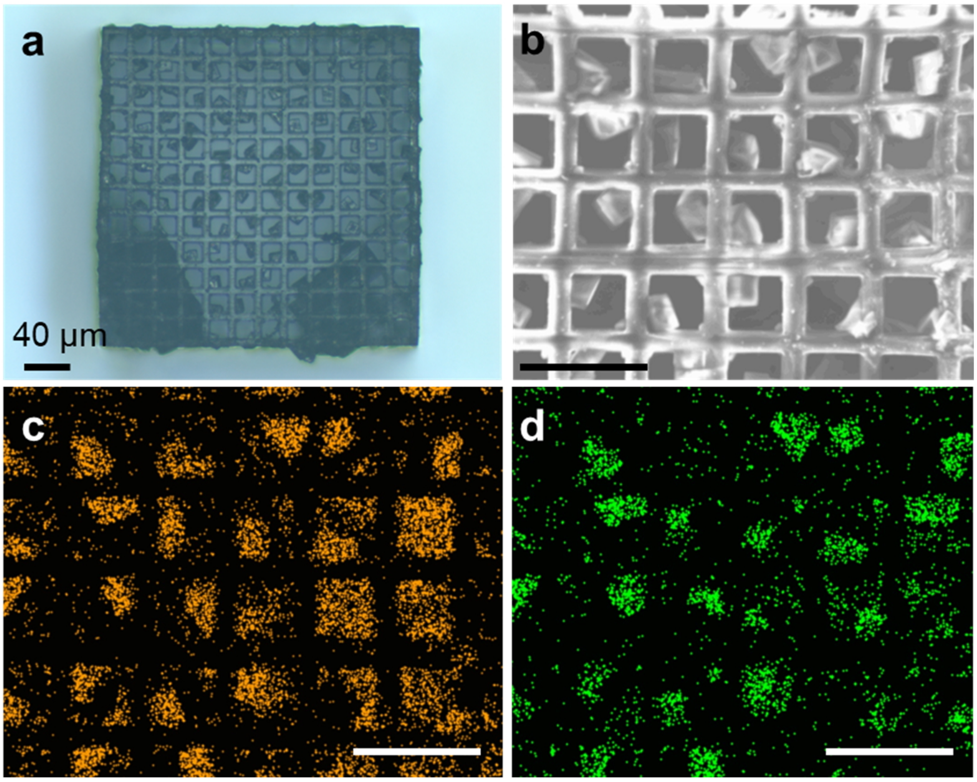

Supplementary Figure 10 The formation of NaCl crystals in micro-scaffolds during the immersion/retraction process. a, b, Optical image (**a**) and SEM image (**b**) of a microscaffold treated in 90% IPA/H_2O solution for 600 immersion/retraction cycles. **c, d,** EDS mappings of Cl (**c**) and Na (**d**) elements distribution correspond to the sample of (**b**).

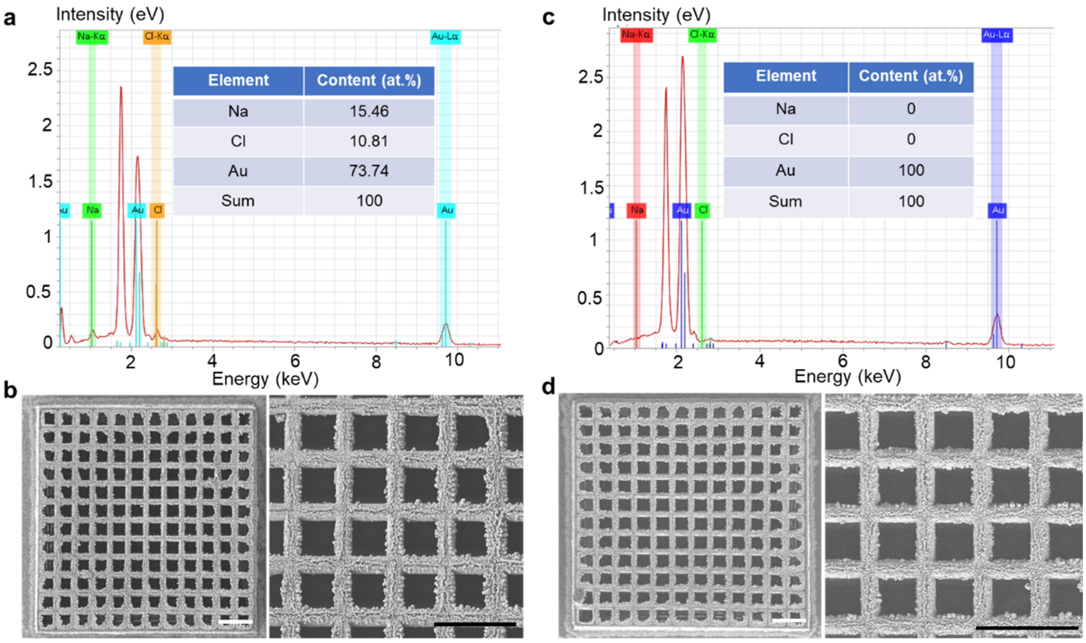

Supplementary Figure 11 Removable NaCl crystals on the nanomaterial-coated micro-scaffolds. a, b, EDS spectrum (**a**) and SEM images (**b**) of a microscaffold coated with Au NPs before the remove of NaCl crystals. **c, d,** EDS spectrum (**c**) and SEM images (**d**) of a microscaffold coated with Au NPs after the remove of NaCl crystals. The samples are treated in 90% IPA/H_2O solutions with ≈1.2 mg mL^{-1} Au NP and 0.01 mM PBS for 600 immersion/retraction cycles, and then immersed in water for 10 min to remove NaCl. Scale bars in (**b, d**) are 40 μm.

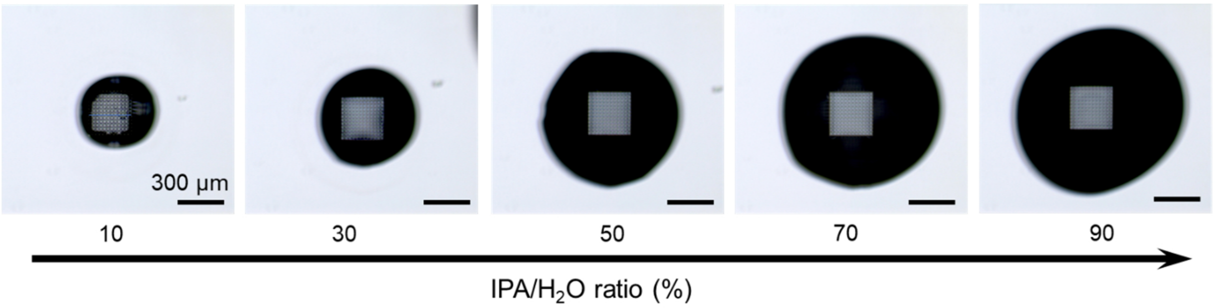

Supplementary Figure 12 Optical images of a microscaffold trapping solutions with 0.01 mM PBS solutions and different IPA/H_2O ratios (10%, 30%, 50%, 70%, and 90%). These experiments are performed in a Zeiss, Axio Imager 2 microscope. 30 µL solution is first dropped on the substrate to cover the micro-scaffold, the excess solution is then removed by a pipette. These images are all captured at the time when the trapped solution is separated from the bulk solution. The eventual volume of trapped solution by a microscaffold is obtained by averaging the results of 4 dependent tests.

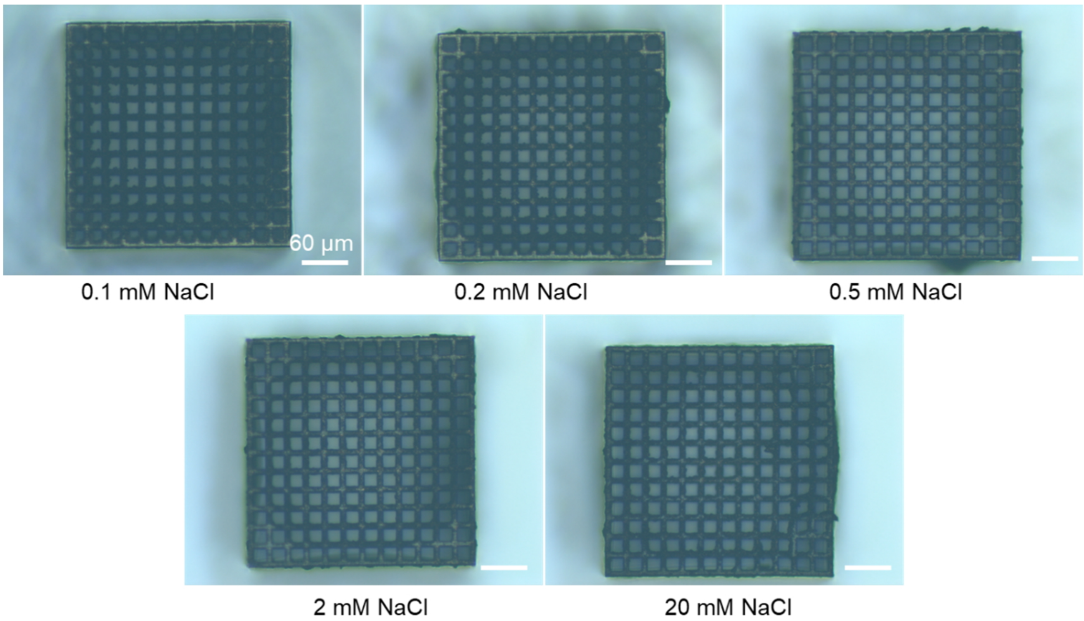

Supplementary Figure 13 Optical images of micro-scaffolds coated with Au NPs in solutions with different NaCl concentrations. The samples are treated in 90% IPA/H_2O solutions with ≈ 1.2 mg mL^{-1} Au NPs and 0.01 mM PBS for 600 immersion/retraction cycles, and then immersed in water for 10 min to remove NaCl.

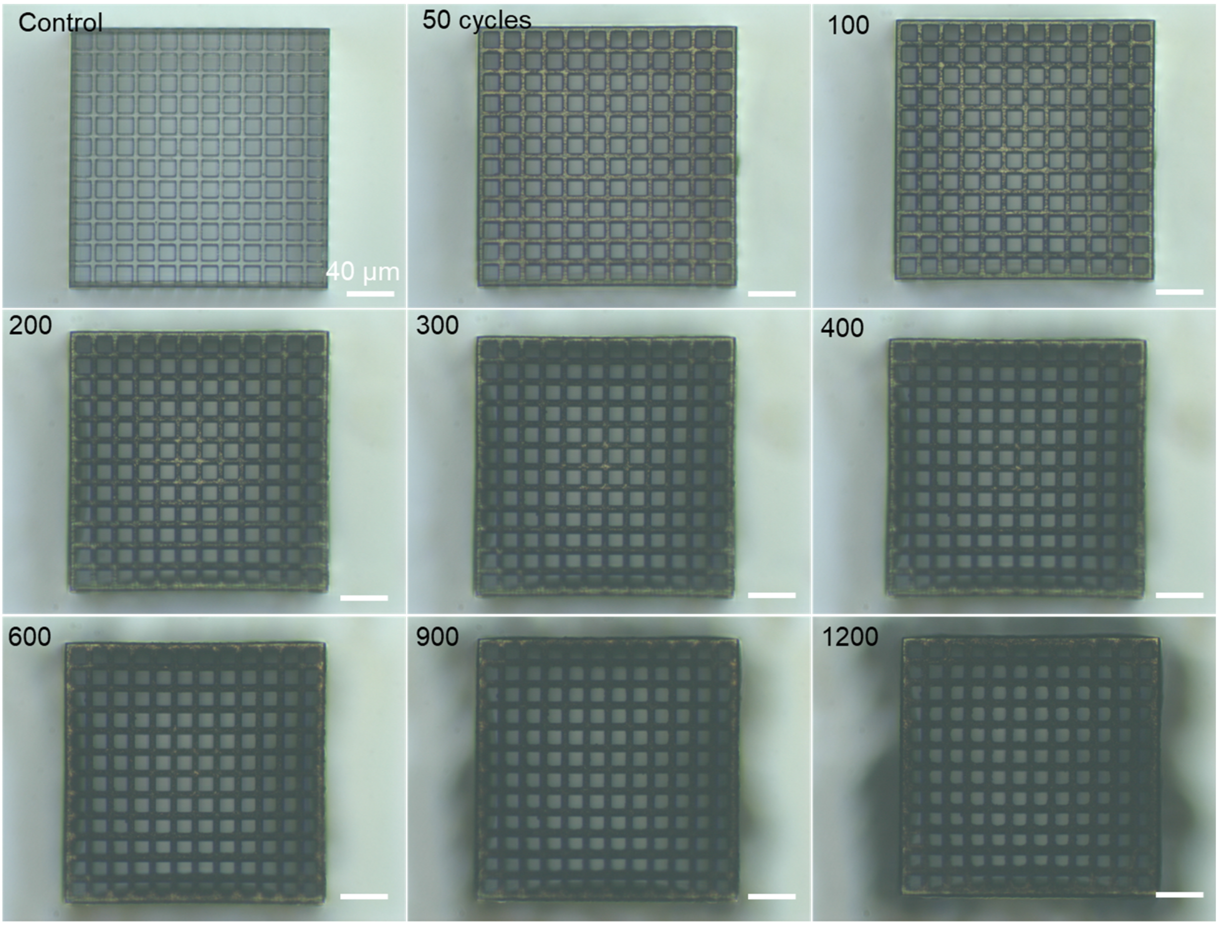

Supplementary Figure 14 Optical images of micro-scaffolds coated with Au NPs for different immersion/retraction cycles. The solution is comprised of ≈1.2 mg mL^{-1} Au NP, 0.01mM PBS, and 90% IPA/H_2O.

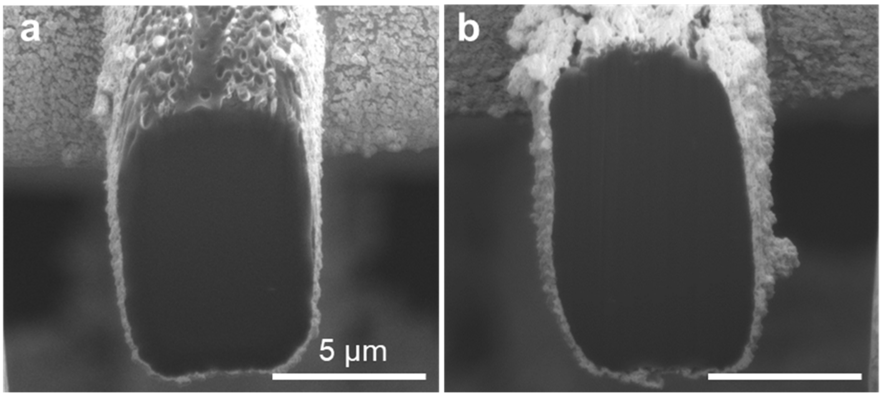

Supplementary Figure 15 a, b, Cross-section SEM images of a FIB-cut microscaffold beam coated with Au NPs for 600 (a) and 1200 (b) immersion/retraction cycles.

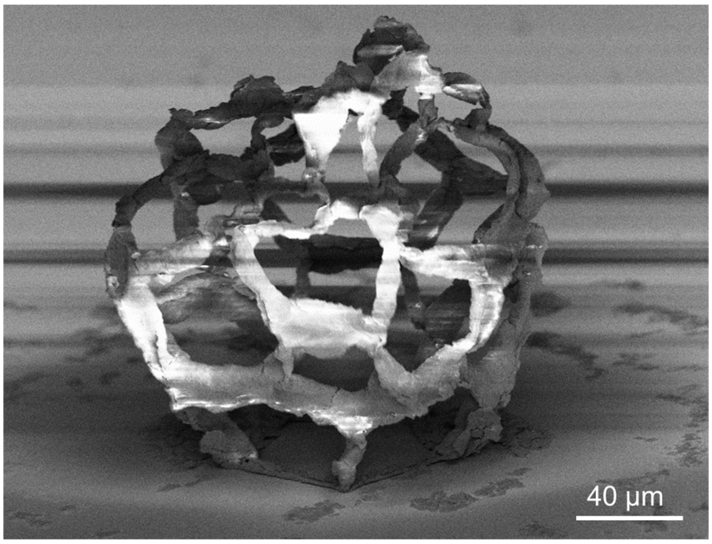

Supplementary Figure 16 SEM image of a 3D hollow microsphere coated with Fe_3O_4 NPs in solution with 0.2 mg mL^{-1} Fe_3O_4 and 0.01 mM PBS for 150 immersion/retraction cycles after annealing treatment (600 °C for 2 h).

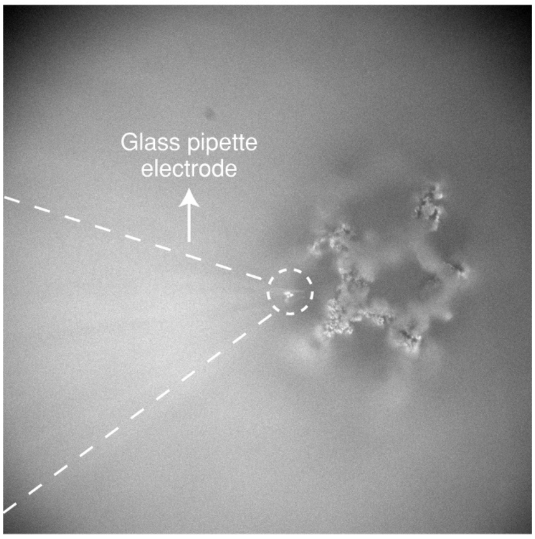

Supplementary Figure 17 Optical image of a glass pipette electrode approaching a 600 °C-annealed Fe_3O_4-$BaTiO_3$ 3D hollow microsphere.

Supplementary Table 1 Comparison among different micro/nanofabrication technologies of multi-material.

		**Resolution**	**Roughness**	**Mechanical performance#**	**Mass loading of desired material**	**Material versatility**	**Printing speed**	**Distribution of desired material after printing**	**Ref.**
**Direct-laser writing**	Cross-linkable metal coordinated compounds,	Sub-200 nm	/	≈20 MPa, after photoresist removed by sintering[1]	<50%	Limited by specific chemistries	1 -50,000 $\mu m\ s^{-1}$	Chemically bonded with other heterogeneous materials	1,2
	Cross-linkable colloidal nanocrystals		≈12 nm[3]	>2 GPa, sintered at 700 ºC[4]	≈90%	Colloids (size< 30 nm) with specific surface ligands, e.g. C-H, Zn-S	1 - 50 $\mu m\ s^{-1}$	Assembly of desired nanocrystals	3,4
	Physically blending with nanomaterials		/	4.5 GPa, sintered at 1200 °C[5]	<50%	Transparent nanomaterial with size< 30 nm (silica and some ceramics)	1 -50,000 $\mu m\ s^{-1}$	Embedded in the polymer matrix	5,6
**Template-assisted fabrication**	Adsorption via hydrogen bonding	≈ 20 nm	≈5 nm	/	≈60%	Water dispensable nanomaterials	Up to 100,000 $\mu m\ s^{-1}$ for the template printing, the deposition time of desired materials ranges from minutes to a few hours	Embedded in the polymer matrix	7
	Electrostatic adsorption	≈100 nm*	/	/	Single nanoparticle layer with loosely discrete distribution	Limited by the surface group of nanomaterials, whose surface charge must be opposite to the template		Deposited on the surface of the polymer template	8
	Chemical coating		/	/	Depends on the amount of chemically active position of template surface	Limited by specific chemical reactions, e.g. electroless plating of metals			9
	Atomic layer deposition		≈1 nm[10,11]	0.28-2.56 GPa, after polymer removed[12-14]	Accumulate along the deposition time	Metal, metal oxide, nitride, etc.			12-14
	Physical deposition (sputtering, evaporating deposition, etc.)		≈1 nm[15]	/		Metal, metal oxide			16
	Capillary trapping assisted deposition **(this work)**		≈46 nm	Material- and structure-dependent	Accumulate along the immersion/retraction cycles	Broader material applicability (polymeric and non-polymeric nanomaterials) (size: 5-500 nm, Without considering specific chemistries)			

#: Engineering compressive strength.
/: No related data reported in literatures.
*: It depends on the resolution of 2PP-printed scaffold, which is around 100 nm[17].

Supplementary Table 2 The physicochemical properties of nanomaterials used in this work.

**Nanomaterials**	**Size (nm)**	**Surface group**	**Dispersed solvent**
Au NPs	5 - 400	/	0.1 mM PBS
Au nanorods	25×60	CTAB	water
Ag NPs	<100	/	powder
Pt NPs	<50	/	powder
Fe_3O_4 NPs	30	Amine, PEG, or carboxylic acid	water
Fe_3O_4 NPs	5	/	Toluene
$BaTiO_3$	50, 280	/	powder
PS	500	Red fluorescence	water
PLGA	100	Orange Fluorescent	powder
diamond	< 10	/	powder
$NaYF_4$ up conversion NPs	30-35	Polyacryclic acid (PAA)	water
Cu NPs	25	/	powder
TiO_2 Nps	25-300	/	powder
Au NPs	50	Alkyne	water
Au NPs	70	azide	water
CdSe/ZnS Quantum dots	/	carboxylic acid	water

Supplementary Table 3 Experimental parameters for coating various materials.

**Nanomaterials**	**Concentration**	**Immersion-retraction cycles**
Au NPs	$\approx$1.2 mg mL^{-1}	600
Au nanorods	$\approx$0.13 mg mL^{-1}	900
Ag NPs	0.3 mg mL^{-1}	300
Pt NPs	0.5 mg mL^{-1}	300
Fe_3O_4 NPs	0.2 mg mL^{-1}	300
$BaTiO_3$	2 mg mL^{-1}	100
PS	0.0025 wt.%	300
PLGA	0.125 mg mL^{-1}	400
Quantum dots	0.05 mg mL^{-1}	900

Supplementary References

1 Vyatskikh, A. *et al.* Additive manufacturing of 3D nano-architected metals. *Nat. Commun.* **9**, 593 (2018).

2 Liu, J. *et al.* 3D printing nano-architected semiconductors based on versatile and customizable metal-bound composite photoresins. *Adv. Mater. Technol.* **7**, 2101230 (2022).

3 Liu, S.-F. *et al.* 3D nanoprinting of semiconductor quantum dots by photoexcitation-induced chemical bonding. *Science* **377**, 1112-1116 (2022).

4 Li, F. *et al.* 3D printing of inorganic nanomaterials by photochemically bonding colloidal nanocrystals. *Science* **381**, 1468-1474 (2023).

5 Sänger, J. C. *et al.* Entering a new dimension in powder processing for advanced ceramics shaping. *Adv. Mater.* **35**, 2208653 (2023).

6 Wen, X. *et al.* 3D-printed silica with nanoscale resolution. *Nat. Mater.* **20**, 1506-1511 (2021).

7 Han, F. *et al.* Three-dimensional nanofabrication via ultrafast laser patterning and kinetically regulated material assembly. *Science* **378**, 1325-1331 (2022).

8 Issa, A. *et al.* One strategy for nanoparticle assembly onto 1D, 2D, and 3D polymer micro and nanostructures. *ACS Appl. Mater. Interfaces* **13**, 41846-41856 (2021).

9 Formanek, F. *et al.* Three-dimensional fabrication of metallic nanostructures over large areas by two-photon polymerization. *Opt. Express* **14**, 800-809 (2006).

10 Elam, J., Sechrist, Z. & George, S. ZnO/Al_2O_3 nanolaminates fabricated by atomic layer deposition: growth and surface roughness measurements. *Thin Solid Films* **414**, 43-55 (2002).

11 Myers, T. J. *et al.* Smoothing surface roughness using Al_2O_3 atomic layer deposition. *Appl. Surf. Sci.* **569**, 150878 (2021).

12 Bauer, J., Hengsbach, S., Tesari, I., Schwaiger, R. & Kraft, O. High-strength cellular ceramic composites with 3D microarchitecture. *Proc. Natl. Acad. Sci. U. S. A.* **111**, 2453-2458 (2014).

13 Meza, L. R., Das, S. & Greer, J. R. Strong, lightweight, and recoverable three-dimensional ceramic nanolattices. *Science* **345**, 1322-1326 (2014).

14 Meza, L. R. & Greer, J. R. Mechanical characterization of hollow ceramic nanolattices. *J. Mater. Sci.* **49**, 2496-2508 (2014).

15 Yamamoto, M. *et al.* Effect of Au film thickness and surface roughness on room-temperature wafer bonding and wafer-scale vacuum sealing by Au-Au surface activated bonding. *Micromachines* **11**, 454 (2020).

16 Xia, X. *et al.* Electrochemically reconfigurable architected materials. *Nature* **573**, 205-213 (2019).

17 Kawata, S., Sun, H.-B., Tanaka, T. & Takada, K. Finer features for functional microdevices. *Nature* **412**, 697-698 (2001).
