# Supplementary material for: Capillary trapping of various nanomaterials on additively manufactured scaffolds for 3D micro-/nanofabrication
Source: Nat Commun. 2024 Aug 6;15:6693. doi: 10.1038/s41467-024-51086-2 (PMC11303746; doi:10.1038/s41467-024-51086-2)
Supplement: Supplementary file 3 — Description of Additional Supplementary Files [file 41467_2024_51086_MOESM3_ESM.pdf]

Description of Additional Supplementary Files

File Name: Supplementary Movie 1

Description: The evaporation process of Au NPs suspension trapped by a micro-scaffold. The video is speeded up 3 times.

File Name: Supplementary Movie 2

Description: Five different materials are deposited automatically and simultaneously using our custom-built platform. The video is speeded up 4 times.

File Name: Supplementary Movie 3

Description: Compression of a 120 oC annealed microcube assembled with Au NPs.

File Name: Supplementary Movie 4

Description: Compression of a 900 oC annealed Fe3O4 microsphere.

File Name: Supplementary Movie 5

Description: Magnetically controlled rolling of 3D hollow Fe3O4 microspheres (after 600 ℃ annealed) in DI water.
